# Current evidence for directed and supportive investigational therapies against COVID-19

**DOI:** 10.7196/AJTCCM.2020.v26i2.072

**Published:** 2020-04-24

**Authors:** V van Rensburg, V Pillay-Fuentes Lorente, E Decloedt

**Affiliations:** ^1^ Division of Clinical Pharmacology, Department of Medicine, Faculty of Medicine and Health Sciences, Stellenbosch University, Cape Town, South Africa; ^2^ Division of Clinical Pharmacology, Department of Medicine, Faculty of Medicine and Health Sciences, Stellenbosch University, Cape Town, South Africa; ^3^ Division of Clinical Pharmacology, Department of Medicine, Faculty of Medicine and Health Sciences, Stellenbosch University, Cape Town, South Africa

**Keywords:** covid-19, coronavirus

## Abstract

Coronavirus disease 2019 (COVID-19) is a global health crisis. There is currently a great need for effective and safe therapies directed at the disease, but no drugs are presently registered for use in COVID-19. Several directed therapies have been proposed, and most are still in clinical trials. Currently available published, peer-reviewed results mostly involve small sample sizes with study limitations restricting the interpretation of the findings. Many trials currently published also do not have a control group, limiting the interpretation of the effect of the intervention. Investigational directed therapies as well as investigational supportive therapies against COVID-19 are reviewed here. Chloroquine and hydroxychloroquine show promise as directed therapies, but current trial results are conflicting. Lopinavir/ritonavir also shows potential, but was started late in the disease course in most trials. No randomised controlled evidence is currently available for remdesivir and favipiravir. Corticosteroid use is not recommended for directed therapy against COVID-19, and the role of tocilizumab is currently unclear, based on limited evidence. Early initiation of investigational directed therapies may provide benefit in selected patients. The results from larger randomised controlled trials will clarify the place of these therapies in COVID-19 treatment.

*Afr J Thoracic Crit Care Med* 2020;26(2): Published online 30 April 2020. https://doi.org/10.7196/AJTCCM.2020.v26i2.072

The novel coronavirus responsible for the coronavirus disease 2019 (COVID-19) pandemic, severe acute respiratory syndrome coronavirus 2 (SARS-CoV-2), was first identified in Wuhan, China in December 2019.[1] The virus spread from China to many other countries in the world in a matter of weeks, leading the World Health Organization (WHO) to declare COVID-19 a pandemic in March 2020.[2] The clinical presentation is that of mild to moderate disease in 80% of patients,[3] but as of 17 April 2020, more than 130 000 people had died from COVID-19 worldwide, with 2 million infected.[4] Several risk factors for severe disease and death have been identified, including older age (≥65 years old) and certain comorbid conditions including diabetes, chronic cardiac, renal, and liver disease, obesity and immunocompromise.[5] There is currently a great need for effective and safe therapies directed at COVID-19, but there are currently no registered drugs available for the treatment of COVID-19.[6] The generation and dissemination of publications on experimental targeted COVID-19 therapies is occurring at an unprecedentedly rapid pace. The international scientific community and clinicians must, however, guard against the acceptance, propagation and implementation of anecdotal evidence, as well as clinical trial results that are released prematurely. Results from non-peer-reviewed, pre-print or uncontrolled trials must be interpreted via acknowledgment of their weaknesses and limitations. Findings from such trials, if used to guide clinical practice, may cause harm if the methodology, results and patient population are not properly appraised.

The management of milder cases is predominantly directed at symptomatic treatment, and the approach for moderate to severe presentations is mainly based on supplemental oxygen and supportive management.[7-9] Concomitant antibiotics and antivirals may also be used as empirical or definitive therapy for other pathogens.[9] The global clinical need for effective therapy has led to the investigational repurposing of registered drugs, as well as the early-phase testing of new antiviral drugs. Many of these drugs have already been investigated for SARS-CoV-1, Middle East respiratory syndrome (MERS) or Ebola virus, with inconsistent efficacy results.[10,11] There are currently no clear data from randomised controlled clinical trials on the effect of these drugs on COVID-19 outcomes, but several hundred clinical trials are presently registered to investigate their efficacy and safety in humans.[12,13] Below we briefly and narratively summarise the current evidence published in peer-reviewed scientific journals for the listed investigational therapies.

## Investigational directed therapies for COVID-19

### Chloroquine and hydroxychloroquine

Chloroquine (CQ) has been the backbone of malaria treatment and prevention for many decades. It is also used as an immunomodulator for rheumatological conditions such as systemic lupus erythematosus and rheumatoid arthritis (RA).[14] CQ has good *in vitro* activity against SARS-CoV-2.[15] The postulated mechanisms of action in COVID-19 infection are threefold:[16] increasing endosomal pH that inhibits the SARS-CoV-2 spike protein cleavage required for viral/endosomal fusion after entry; interference with the glycosylation of cellular receptors (possibly through modification of angiotensin-converting enzyme 2 (ACE2), purported to be involved with viral entry);[11,17] and immunomodulation. Multiple trials across the globe are currently underway to assess the efficacy of CQ for the treatment and prevention of COVID-19, but no published, peer-reviewed results are available at the time of writing.[12,13,16,18]

Similarly to CQ, hydroxychloroquine (HCQ) has *in vitro* activity against SARS-CoV-2.[15] HCQ was found to be more potent than CQ at inhibiting SARS-CoV-2 *in vitro,*[19] and is expected to have a similar mechanism of antiviral action to CQ.[20] HCQ is generally regarded as having fewer long-term adverse effects than CQ at equivalent doses, most notably retinopathy.[20,21] The mechanisms underlying this difference are currently unknown, but may be related to differences in drug accumulation, or the reduced propensity of HCQ to bind to eye tissues, compared with CQ.[22] Cardiotoxicity, especially QT-prolongation, is, however, a significant risk with both drugs, even early in the treatment course.[23] The mechanism of drug-induced QT prolongation is through inhibition of the HERG/Kv11.1 potassium channels in the heart.[23] Inhibiting these channels increases the cardiac action potential duration, prolonging the QT interval and increasing the risk for *torsades de pointes*.[24] The risk of QT-prolongation is further compounded in patients with underlying cardiac disease or cardiac risk factors, and with the administration of concomitant QT-prolonging medications or enzyme inhibitors.[23,25] The pervasive use of certain tuberculosis drugs and HIV antiretroviral therapy in the southern African setting may further increase the QT-prolongation risk.[26] Several risk-stratifying tools are currently available for drugs with QT-prolonging potential. A newly launched platform, MedSafety Scan (https://medsafetyscan.org), has been made available online to guide clinicians on risk and clinical decision-making when they input patients' QT-prolonging drugs and selected clinical factors. The risk of CQ and HCQ to cause QT prolongation is based on the well-known CredibleMeds (https://crediblemeds.org) database.

The currently available evidence on the use of HCQ in COVID-19 comes from four published, peer-reviewed trials. Gautret *et al.*[27] suggested in a small, open-label, non-randomised trial that HCQ together with azithromycin (AZM) may be more effective in reducing viral shedding by day 6 post-treatment than HCQ alone, or controls. Of note is that the effect of AZM was not prospectively assessed with HCQ, as the AZM was only given to some patients as part of standard of care if the clinical presentation warranted it. The association of AZM and HCQ in the reduction of viral shedding was therefore made after the fact. Some limitations of this trial were the small sample size and lack of power (only six participants received HCQ and AZM), no reporting of clinical outcomes and exclusion of participants who died or were sent to the intensive care unit (ICU). The authors commented that the addition of AZM to HCQ may increase the risk of QT-prolongation, but that the potential antiviral activity of AZM and the synergistic effect with HCQ may justify the combination on a case-by-case basis. Gautret *et al.*[28] published a follow-on uncontrolled study with a larger sample size ($n$=80). Primary endpoints were the need for oxygen therapy or transfer to the ICU after at least 3 days of treatment, contagiousness as assessed by viral real-time reverse-transcriptase polymerase chain reaction (RT-PCR) and culture, and length of stay in the study ward. Dosages used were oral HCQ sulphate 200 mg three times a day for 10 days, combined with AZM 500 mg on day 1, followed by 250 mg per day for the next 4 days. HCQ with AZM was not started in patients in whom the cardiac risk was deemed too high. The primary endpoint was reached in 18.8% of participants, who required oxygen therapy or transfer to the ICU. Viral RT-PCR was negative in 83% at day 7, and 100% at day 12 post treatment. Viral cultures were negative in 97.5% of participants at day 5. Sixty-five of the 80 participants were discharged from the study ward during the trial period, with a mean time from initiation to discharge of 4.1 days. The findings of this trial are difficult to interpret, as participants were not randomised, nor was there a control group. Study limitations further include that most participants (92%) were considered low risk for clinical deterioration at enrolment, and this low risk continued until discharge in most (93.8%). Four asymptomatic patients were also included in the trial, and more than three-quarters of participants were younger than the high-risk age group of ≥65 years old.[5] The majority of participants had a low risk for clinical deterioration, and the observation of negative RT-PCR tests in 83% of participants by day 7 after treatment initiation – therefore at a median of day 12 after symptom onset – could well be in keeping with the natural course of the disease.[3,31]

In response to the first publication by Gautret *et al*.,[27] Molina *et al.*[30] replicated the design and dosages of HCQ and AZM in their own trial to assess whether similar findings were observed. They prospectively enrolled 11 consecutively hospitalised patients with severe COVID-19. Paired RT-PCR results were available in 10 participants, with 8 still testing positive at days 5 - 6 after treatment initiation. The authors conclude that their findings stand in contrast to those reported by Gautret *et al*.,[27] and cast doubt on the strong antiviral efficacy of the combination of HCQ and AZM.[30] Limitations of this trial include a small sample size, no control group and limited information on clinical outcomes.

A Chinese study evaluated 30 participants randomised to receive HCQ sulphate 400 mg once a day for 5 days with standard of care ($n$=15), or standard of care alone ($n$=15).[31] The study endpoints were virological clearance by day 7, or death. The authors found no difference between the two groups for virological clearance (86.7% in the interventional group v. 93.3% in the standard of care group; $p$>0.05). None of the participants died during the 2-week follow-up period.[31]

In summary, trials assessing HCQ and CQ in COVID-19 to date have mostly been uncontrolled, and have shown conflicting results. Owing to methodological limitations, the results are difficult to interpret, and there are currently insufficient clinical data to recommend either for or against the use of CQ and HCQ in COVID-19 outside of the clinical trial setting.[32]

## Lopinavir/ritonavir (with or without interferon)

Lopinavir and ritonavir are both protease inhibitors. Ritonavir is used in combination with lopinavir to increase lopinavir plasma concentrations by inhibiting the enzymatic metabolism of lopinavir.[33] The rationale for the use of lopinavir/ritonavir (LPV/r) in COVID-19 stems from its *in vitro* activity against SARS-CoV-1,[34] as well as from a retrospective, multicentre cohort study evaluating LPV/r as early treatment in SARS-CoV-1, which demonstrated decreased mortality and intubation rates.[35] Numerous case reports and case series have shown successful management of COVID-19 patients with LPV/r.[36-41] Nevertheless, some patients on LPV/r still progressed to severe disease, although in most such cases LPV/r was only started late in the disease course. It is therefore unclear whether the observed effect was due to the intervention or to the natural course of the disease. In a retrospective cohort study of 33 patients receiving LPV/r 400/100 mg twice a day, all patients tested negative with RT-PCR by day 14.[42] Wan *et al*.[43] reported the clinical features and treatment of COVID-19 in a case series of 135 patients in China. They reported that LPV/r and interferon administered to all patients in early-stage disease resulted in the discharge of 15 patients and the death of 1 patient at the end of the 16-day study period. A total of 119 patients were still hospitalised at the end of the study period. The doses of LPV/r, and the doses and type of interferon used, were not stated. *In vitro* studies of interferon have demonstrated inhibition of SARS-CoV-1 infection, but only in combination with other antiviral agents.[44] Several clinical trials are currently evaluating various treatment combinations with interferon.[12,13] The most robust evidence for LPV/r in SARS-CoV-2 currently comes from a Chinese randomised controlled trial that evaluated its efficacy and safety.[45] The primary outcome was the time to clinical improvement. One hundred and ninety-nine participants were individually randomised to LPV/r ($n$=99) at a dose of 400/100 mg twice a day with standard of care, or standard of care alone ($n$=100), for 14 days. The results showed that the time to clinical improvement between the two groups was similar, with a median of 16 days for both (hazard ratio for clinical improvement 1.31; 95% confidence interval 0.95 - 1.80; $p$=0.09).[47] Recorded adverse events included nausea, vomiting and diarrhoea, all of which were more common in the LPV/r group than the standard of care group. Four gastrointestinal-related serious adverse events reported in the LPV/r group were deemed to be related to LPV/r. Limitations included the open-label design of the trial, the initiation of investigational treatment late in the disease course (a median of 13 days after symptom onset) and the fact that both groups were heterogeneous and received various additional treatments. In summary, LPV/r does not currently have robust evidence for use in COVID-19, but the mostly conflicting results from currently available data should be interpreted in light of the study designs and timing of LPV/r in the disease course. At the time of writing, LPV/r is not recommended for use, other than as part of clinical trials.[32]

### Remdesivir

Remdesivir is in early-phase drug development, and currently being investigated for COVID-19 therapy. Remdesivir was originally developed for use against the Ebola virus in 2015,[46] and has shown promising *in vitro* activity against SARS-CoV-2.[15] It acts as a nucleotide analogue, causing premature termination of viral RNA replication by inhibiting RNA polymerase.[47] Although remdesivir is not yet registered by any medicines regulatory authority for human use, its safety profile was established in a randomised controlled trial during the Ebola virus outbreak in the Democratic Republic of Congo.[46] Several clinical trials are currently registered for the investigation of remdesivir,[12,13] but no robust results are yet available. Remdesivir has, however, been approved for compassionate use in several countries, and a recent preliminary report described the outcomes in a cohort of patients hospitalised for severe COVID-19 who were treated with remdesivir on a compassionate-use basis.[48] The authors described the outcomes of 53 participants, and reported that after a median follow-up of 18 days, 68% of the participants had an improvement in terms of their required oxygen support. Of 34 participants who initially required mechanical ventilation, 9 remained ventilated, 3 were switched to non-invasive ventilation, 8 were weaned off oxygen and 8 were discharged. Six participants who were initially ventilated died, and the overall mortality rate was 13%. A significant limitation of this report is the protracted duration of symptoms before remdesivir was started (median (interquartile range) 12 (9 - 15) days). Additionally, the absence of a control group limits the interpretation of the effect of remdesivir. Concomitant therapies and viral loads were also not reported. In summary, there are currently insufficient clinical data available to recommend either for or against the use of remdesivir in COVID-19, outside of clinical trials.[32]

### Favipiravir

Favipiravir is an antiviral drug approved for the management of influenza in Japan.[49] It is currently being evaluated in clinical trials for the management of COVID-19.[12] It is a nucleic acid analogue that is incorporated into viral RNA to affect viral replication and possibly RNA polymerase.[50] At the time of writing, there were no published peer-reviewed trials or case studies evaluating favipiravir in COVID-19, and its use is not currently recommended outside of clinical trials.

## Investigational supportive therapies for COVID-19

### Tocilizumab

Tocilizumab is a monoclonal antibody used for the treatment of several rheumatic conditions, including RA and juvenile idiopathic arthritis.[11,51] The US Food and Drug Administration additionally approved tocilizumab in 2017 for the treatment of cytokine release syndrome (CRS, also known as cytokine storm) caused by chimeric antigen receptor T-cell therapy, an immunomodulatory approach used in oncology.[52] Tocilizumab is a recombinant humanised monoclonal antibody against the interleukin 6 (IL-6) receptor. It binds both soluble and membrane-bound IL-6 receptors, inhibiting IL-6 signal transduction.[52] Patients with severe disease during the previous outbreaks of SARS-CoV-1[53] and MERS[54] were found to have elevated concentrations of IL-6. Patients who died due to COVID-19 were also found to have increased markers for CRS, including raised IL-6.[40] It is therefore postulated that the current severe organ dysfunction observed in COVID-19 may be due to CRS, and that IL-6 inhibitors such as tocilizumab may attenuate this immune reaction.[11,55,56]

A retrospective observational study from a single centre in China reported on the use of tocilizumab in 15 COVID-19 patients.[57] The study population was older patients, aged between 62 and 80 years. Most patients were severely or critically ill, and over half received a course of methylprednisolone. One-third of the patients (5/15) received two or more doses of tocilizumab. The authors report increasing IL-6 concentrations after tocilizumab initiation followed by a decrease in most patients who improved clinically. Five patients died or had disease aggravation, with IL-6 concentrations increasing in all five. Limitations include the small sample size and the non-reporting of the duration of symptoms before treatment was started, or whether other investigational therapy was given as well. Several case reports and a case series have also been published describing the effect of tocilizumab in patients with various underlying conditions.[58-61] All showed improvement, but these reports included patients who were started on tocilizumab after inadequate response to standard of care. It is unclear what the effect of tocilizumab was in these cases, as it was started late in the disease course and the clinical improvement may have been in keeping with the natural course of the disease. Numerous randomised trials are currently underway to evaluate tocilizumab in COVID-19.[12,13]

### Corticosteroids

The WHO advises against the use of corticosteroids in the management of targeted COVID-19 owing to potential harmful effects.[9] This is largely based on previous experiences of corticosteroid therapy in influenza and MERS-CoV. A systematic review of treatment options in SARS-CoV-1 infection included corticosteroids' effects on mortality, *in vitro* inhibition of SARS viral replication and acute respiratory distress syndrome.[62] The study concluded that the evidence for the effects of corticosteroids in the management of SARS-CoV-1 was either inconclusive or showed that they conferred possible harm.[62] However, the studies included were of poor-quality evidence. A Cochrane review[63] of observational studies of corticosteroids as adjunctive therapy in the treatment of influenza found an increased risk of mortality. The included studies demonstrated significant heterogeneity across the group as a whole, and the quality of evidence was graded as low.[63]

A subsequent observational cohort study investigating influenza adjusted for baseline and time-dependent characteristics, and matching treatment groups according to propensity scores, found no association between the use of corticosteroids and mortality in influenza.[64] In MERS-CoV, there was an association between delayed viral clearance and the use of corticosteroids, but no association with 90-day mortality.[64] The major limitation of this study was the retrospective observational design, with possible confounding by indication. Nevertheless, owing to the possibility of potential harm by corticosteroids in viral infections, the WHO recommends against the routine use of corticosteroids in COVID-19 patients, unless used with caution for other indications where there are supportive data, such as sepsis and acute exacerbations of asthma and chronic obstructive pulmonary disease, or as part of clinical trials.[9] Patients infected with COVID-19 subsequently require a risk-benefit assessment prior to the use of corticosteroids. At the time of writing there were no published, peer-reviewed studies on the use of corticosteroids in COVID-19, but several clinical trials are underway.[12,13]

## Timing of investigational directed therapies for COVID-19

Several of the above trials have shown that results from investigational therapies that were started late in the disease course are difficult to interpret, as the outcomes observed may have been masked by irreversible systemic injury (no difference compared with standard of care), or natural course of the disease (improvement regardless of the therapy). Clinical trials will subsequently require large sample sizes to determine if the investigational directed therapy is in fact effective for smaller treatment effects later in the disease course. In addition, investigational supportive therapies such as tocilizumab may be more effective when given later in the disease course with more severe immunopathology, acting to decrease organ damage in the presence of CRS. Conversely, some therapies may be more beneficial when given early. Oseltamivir, an antiviral drug used for influenza virus infection, has been shown to be more effective when given 48 - 72 hours after symptom onset.[65] The rationale is that influenza viral replication peaks at 24 - 72 hours after symptom onset,[66] and viral replication inhibitors such as oseltamivir should be given no later than at the peak of viral replication. Drugs that purportedly inhibit SARS-CoV-2 replication (such as the investigational antivirals) or viral entry and replication (CQ and HCQ) may therefore be more effective when given earlier in the COVID-19 disease course. Patients who may benefit from earlier treatment initiation include those with confirmed COVID-19 who do not require hospitalisation, but have significant risk factors.

## Conclusion

There is a pressing need globally to find effective treatment options for COVID-19. Current strategies to curtail the pandemic are aimed at infection prevention and control, and supportive management of those with infections of varying severity. The hope of effective and safe large-scale preventive strategies such as vaccines seems to still be several months away. In the interim, numerous clinical trials are ongoing to investigate various repurposed and experimental drugs, but no published, peer-reviewed trial to date has provided clear evidence of significant benefit with permissible safety for any drug for COVID-19. In selected patients, early initiation of investigational directed therapy may be of benefit due to reduced viral replication and/or entry. Amid the turmoil of the current pandemic, investigators and sponsors should as far as possible conduct COVID-19 trials in a way that allows for clear interpretation of the results, and the scientific community should continue to uphold the rigorous principles of evidence-based medicine and proper conduct and reporting of clinical trials.

**Acknowledgements.** None.
**Author contributions.** Equal contributions.
**Funding.** None.
**Conflicts of interest.** None.

1. Zhu N, Zhang D, Wang W, et al. A novel coronavirus from patients with pneumonia in China, 2019. N Engl J Med 2020;382(8):727-733. https://doi.org/10.1056/NEJMoa2001017
2. World Health Organization. WHO Director-General's opening remarks at the media briefing on COVID-19 – 11 March 2020. Geneva: WHO, 2020. https://www.who.int/dg/speeches/detail/who-director-general-s-opening-remarks-at-the-media-briefing-on-covid-19---11-march-2020 (accessed 15 April 2020).
3. World Health Organization. Report of the WHO-China joint mission on coronavirus disease 2019 (COVID-19). Geneva: WHO, 2020. https://www.who.int/docs/default-source/coronaviruse/who-china-joint-mission-on-covid-19-final-report.pdf (accessed 1 April 2020).
4. World Health Organization. Coronavirus disease 2019 (COVID-19) Situation Report – 87. Geneva: WHO, 2020. https://www.who.int/docs/default-source/coronaviruse/situation-reports/20200416-sitrep-87-covid-19.pdf?sfvrsn=9523115a_2 (accessed 17 April 2020).
5. Centers for Disease Control and Prevention. People who are at higher risk for severe illness. Hyattsville, MD: CDC, 2020. https://www.cdc.gov/coronavirus/2019-ncov/need-extra-precautions/people-at-higher-risk.html (accessed 16 April 2020).
6. World Health Organization. Off-label use of medicines for COVID-19. Geneva: WHO, 2020. https://www.who.int/news-room/commentaries/detail/off-label-use-of-medicines-for-covid-19 (accessed 15 April 2020).
7. National Department of Health, South Africa. Clinical management of suspected or confirmed COVID-19 disease. Pretoria: NDOH, 2020. https://j9z5g3w2.stackpathcdn.com/wp-content/uploads/2020/03/Clinical-Management-of-COVID-19-disease_Version-3_27March2020.pdf (accessed 15 April 2020).
8. Centers for Disease Control and Prevention. Therapeutic options for COVID-19 patients. Hyattsville, MD: CDC, 2020. https://www.cdc.gov/coronavirus/2019-ncov/hcp/therapeutic-options.html (accessed 23 March 2020).

9. World Health Organization. Clinical management of severe acute respiratory infection when COVID-19 is suspected. Geneva: WHO, 2020. https://www.who.int/publications-detail/clinical-management-of-severe-acute-respiratory-infection-when-novel-coronavirus-(ncov)-infection-is-suspected (accessed 15 April 2020).
10. Martinez MA. Compounds with therapeutic potential against novel respiratory 2019 coronavirus. Antimicrob Agents Chemother 2020;64(5):e00399-20. https://doi.org/10.1128/aac.00399-20
11. Sanders JM, Monogue ML, Jodlowski TZ, et al. Pharmacologic treatments for coronavirus disease 2019 (COVID-19). J Am Med Assoc 2020;(epub ahead of print). https://doi.org/10.1001/jama.2020.6019
12. ClinicalTrials.gov. Bethesda (MD): National Library of Medicine (USA), 2020. https://clinicaltrials.gov (accessed 15 April 2020).
13. EU Clinical Trials Register. Amsterdam: European Union, 2020. https://www.clinicaltrialsregister.eu (accessed 15 April 2020).
14. Touret F, de Lamballerie X. Of chloroquine and COVID-19. Antiviral Res 2020;177:104762. https://doi.org/10.1016/j.antiviral.2020.104762
15. Wang M, Cao R, Zhang L, et al. Remdesivir and chloroquine effectively inhibit the recently emerged novel coronavirus (2019-nCoV) *in vitro*. Cell Res 2020;30(3):269-271. https://doi.org/10.1038/s41422-020-0282-0
16. Gao J, Tian Z, Yang X. Breakthrough: Chloroquine phosphate has shown apparent efficacy in treatment of COVID-19 associated pneumonia in clinical studies. Biosci Trends 2020;14(1):72-73. https://doi.org/10.5582/bst.2020.01047
17. Zhang H, Penninger JM, Li Y, et al. Angiotensin-converting enzyme 2 (ACE2) as a SARS-CoV-2 receptor: Molecular mechanisms and potential therapeutic target. Intensive Care Med 2020(46):586-590. https://doi.org/10.1007/s00134-020-05985-9
18. Cortegiani A, Ingoglia G, Ippolito M, et al. A systematic review on the efficacy and safety of chloroquine for the treatment of COVID-19. J Crit Care 2020;(epub ahead of print). https://doi.org/10.1016/j.jcrc.2020.03.005
19. Yao X, Ye F, Zhang M, et al. *In vitro* antiviral activity and projection of optimized dosing design of hydroxychloroquine for the treatment of severe acute respiratory syndrome coronavirus 2 (SARS-CoV-2). Clin Infect Dis 2020:ciaa237. https://doi.org/10.1093/cid/ciaa237
20. Liu J, Cao R, Xu M, et al. Hydroxychloroquine, a less toxic derivative of chloroquine, is effective in inhibiting SARS-CoV-2 infection *in vitro*. Cell Discov 2020;6(1):1-4. https://doi.org/10.1038/s41421-020-0156-0
21. Schrezenmeier E, Dörner T. Mechanisms of action of hydroxychloroquine and chloroquine: Implications for rheumatology. Nat Rev Rheumatol 2020;16(3):155-166. https://doi.org/10.1038/s41584-020-0372-x
22. Yam J, Kwok A. Ocular toxicity of hydroxychloroquine. Hong Kong Med J 2006;12(4):294-304.
23. Giudicessi JR, Noseworthy PA, Friedman PA, et al. Urgent guidance for navigating and circumventing the QTc-prolonging and torsadogenic potential of possible pharmacotherapies for coronavirus disease 19 (COVID-19). Mayo Clin Proc 2020;(epub ahead of print). https://doi.org/10.1016/j.mayocp.2020.03.024
24. Nachimuthu S, Assar MD, Schussler JM. Drug-induced QT interval prolongation: Mechanisms and clinical management. Ther Adv Drug Saf 2012;3(5):241-253. https://doi.org/10.1177/2042098612454283
25. Decloedt EH, Reuter H, Allwood B, et al. Benefit v. risk when using chloroquine in patients with severe COVID-19 disease. S Afr Med J 2020;110(5):(epub ahead of print). https://doi.org/10.7196/samj.2020.v110i5.14761
26. Abena PM, Decloedt EH, Bottieau E, et al. Chloroquine and hydroxychloroquine for the prevention or treatment of novel coronavirus disease (COVID-19) in Africa: Caution for inappropriate off-label use in healthcare settings. 2020:tpmd200290(epub ahead of print). https://doi.org/10.4269/ajtmh.20-0290
27. Gautret P, Lagier J-C, Parola P, et al. Hydroxychloroquine and azithromycin as a treatment of COVID-19: Results of an open-label non-randomized clinical trial. Int J Antimicrob Agents 2020:105949(epub ahead of print). https://doi.org/10.1016/j.ijantimicag.2020.105949
28. Gautret P, Lagier J-C, Parola P, et al. Clinical and microbiological effect of a combination of hydroxychloroquine and azithromycin in 80 COVID-19 patients with at least a six-day follow up: A pilot observational study. Travel Med Infect Dis 2020:101663(epub ahead of print). https://doi.org/10.1016/j.tmaid.2020.101663
29. Liu Y, Yan LM, Wan L, et al. Viral dynamics in mild and severe cases of COVID-19. Lancet Infect Dis 2020;(epub ahead of print). https://doi.org/10.1016/S1473-3099(20)30232-2
30. Molina JM, Delaugerre C, le Goff J, et al. No evidence of rapid antiviral clearance or clinical benefit with the combination of hydroxychloroquine and azithromycin in patients with severe COVID-19 infection. Médecine Mal Infect 2020:30085-30088(epub ahead of print). https://doi.org/10.1016/j.medmal.2020.03.006
31. Chen J, Liu D, Liu L, et al. A pilot study of hydroxychloroquine in treatment of patients with common coronavirus disease-19 (COVID-19). J Zhejiang Univ (Med Sci) 2020;49:1-10. https://doi.org/10.3785/j.issn.1008-9292.2020.03.03
32. National Institutes of Health. COVID-19 treatment guidelines: Antiviral therapy. Bethesda, MD: NIH, 2020. https://covid19treatmentguidelines.nih.gov/therapeutic-options-under-investigation/antiviral-therapy (accessed 22 April 2020).
33. Cvetkovic RS, Goa KL. Lopinavir/ritonavir: A review of its use in the management of HIV infection. Drugs 2003;63(8):769-802. https://doi.org/10.2165/00003495-200363080-00004
34. Chu CM, Cheng CC, Hung FN, et al. Role of lopinavir/ritonavir in the treatment of SARS: Initial virological and clinical findings. Thorax 2004;59:252-256. https://doi.org/10.1136/thorax.2003.012658
35. Que TL, Wong VC, Yuen KY. Treatment of severe acute respiratory syndrome with lopinavir/ritonavir: A multicentre retrospective matched cohort study. Hong Kong Med J 2003;9(6):399-406.
36. Kim JY, Choe PG, Oh Y, et al. The first case of 2019 novel coronavirus pneumonia imported into Korea from Wuhan, China: Implication for infection prevention and control measures. J Korean Med Sci 2020;35(5):e61. https://doi.org/10.3346/jkms.2020.35.e61
37. Han W, Quan B, Guo Y, et al. The course of clinical diagnosis and treatment of a case infected with coronavirus disease 2019. J Med Virol 2020;92(5):461-463. https://doi.org/10.1002/jmv.25711
38. Lim J, Jeon S, Shin HY, et al. Case of the index patient who caused tertiary transmission of coronavirus disease 2019 in Korea: The application of lopinavir/ritonavir for the treatment of COVID-19 pneumonia monitored by quantitative RT-PCR. J Korean Med Sci 2020;35(6):e79. https://doi.org/10.3346/jkms.2020.35.e79
39. Wang Z, Chen X, Lu Y, et al. Clinical characteristics and therapeutic procedure for four cases with 2019 novel coronavirus pneumonia receiving combined Chinese and Western medicine treatment. Biosci Trends 2020;14(1):64-68. https://doi.org/10.5582/bst.2020.01030
40. Zhou F, Yu T, Du R, et al. Clinical course and risk factors for mortality of adult inpatients with COVID-19 in Wuhan, China: A retrospective cohort study. Lancet 2020;395:1054-1062. https://doi.org/10.1016/S0140-6736(20)30566-3
41. Young BE, Ong SWX, Kalimuddin S, et al. Epidemiologic features and clinical course of patients infected with SARS-CoV-2 in Singapore. JAMA 2020;(epub ahead of print). https://doi.org/10.1001/jama.2020.3204
42. Deng L, Li C, Zeng Q, et al. Arbidol combined with LPV/r versus LPV/r alone against coronavirus disease 2019: A retrospective cohort study. J Infect 2020;(epub ahead of print). https://doi.org/10.1016/j.jinf.2020.03.002
43. Wan S, Xiang Y, Fang W, et al. Clinical features and treatment of COVID-19 patients in northeast Chongqing. J Med Virol 2020:1-10;(epub ahead of print). https://doi.org/10.1002/jmv.25783
44. Zhang L, Liu Y. Potential interventions for novel coronavirus in China: A systematic review. J Med Virol 2020;92(5):479-490. https://doi.org/10.1002/jmv.25707
45. Cao B, Wang Y, Wen D, et al. A trial of lopinavir-ritonavir in adults hospitalized with severe Covid-19. N Engl J Med 2020;(epub ahead of print). https://doi.org/10.1056/nejmoa2001282
46. Mulangu S, Dodd LE, Davey RT, et al. A randomized, controlled trial of Ebola virus disease therapeutics. N Engl J Med 2019;381(24):2293-2303. https://doi.org/10.1056/NEJMoa1910993
47. Warren TK, Jordan R, Lo MK, et al. Therapeutic efficacy of the small molecule GS-5734 against Ebola virus in rhesus monkeys. Nature 2016;531(7594):381-385. https://doi.org/10.1038/nature17180
48. Grein J, Ohmagari N, Shin D, et al. Compassionate use of remdesivir for patients with severe Covid-19. N Engl J Med 2020;(epub ahead of print). https://doi.org/10.1056/NEJMoa2007016
49. Hayden FG, Shindo N. Influenza virus polymerase inhibitors in clinical development. Curr Opin Infect Dis 2019;32(2):176-186. https://doi.org/10.1097/QCO.0000000000000532
50. Du Y-X, Chen X-P. Favipiravir: Pharmacokinetics and concerns about clinical trials for 2019-nCoV infection. Clin Pharmacol Ther 2020;(epub ahead of print). https://doi.org/10.1002/cpt.1844
51. Tarr G, Hodkinson B, Reuter H. Superheroes in autoimmune warfare: Biologic therapies in current SA practice. S Afr Med J 2014;104(11):787-791. https://doi.org/10.7196/SAMJ.8947
52. Zhang C, Wu Z, Li J-W, et al. The cytokine release syndrome (CRS) of severe COVID-19 and interleukin-6 receptor (IL-6R) antagonist tocilizumab may be the key to reduce the mortality. Int J Antimicrob Agents 2020:105954(epub ahead of print). https://doi.org/10.1016/j.ijantimicag.2020.105954
53. Tseng CTK, Perrone LA, Zhu H. Severe acute respiratory syndrome and the innate immune responses: Modulation of effector cell function without productive infection. J Immunol 2005;174(12):7977-7985. https://doi.org/10.4049/jimmunol.174.12.7977
54. Zheng Y, Wang Q. Bioinformatics analysis on molecular mechanism of ribavirin and interferon-α in treating MERS-CoV. Chinese J Endem 2016;37(2):291-293. https://doi.org/10.3760/cma.j.issn.0254-6450.2016.02.028

55. Mehta P, McAuley DF, Brown M, et al. COVID-19: Consider cytokine storm syndromes and immunosuppression. Lancet 2020;395(10229):1033-1034. https://doi.org/10.1016/S0140-6736(20)30628-0
56. Buonaguro FM, Puzanov I, Ascierto PA. Anti-IL6R role in treatment of COVID-19-related ARDS. J Transl Med 2020;18(1):165. https://doi.org/10.1186/s12967-020-02333-9
57. Luo P, Liu Y, Qiu L, et al. Tocilizumab treatment in COVID-19: A single-center experience. J Med Virol 2020;(epub ahead of print). https://doi.org/10.1002/jmv.25801
58. Zhang X, Song K, Tong F, et al. First case of COVID-19 in a patient with multiple myeloma successfully treated with tocilizumab. Blood Adv 2020;4(7):1307-1310. https://doi.org/10.1182/bloodadvances.2020001907
59. Michot J-M, Albiges L, Chaput N, et al. Tocilizumab, an anti-IL6 receptor antibody, to treat Covid-19-related respiratory failure: A case report. Ann Oncol 2020;(epub ahead of print). https://doi.org/10.1016/j.annonc.2020.03.300
60. De Luna G, Habibi A, Deux JF, et al. Rapid and severe Covid-19 pneumonia with severe acute chest syndrome in a sickle-cell patient successfully treated with tocilizumab. Am J Hematol 2020;(epub ahead of print). https://doi.org/10.1002/ajh.25833
61. Di Giambenedetto S, Ciccullo A, Borghetti A, et al. Off-label use of tocilizumab in patients with SARS-CoV-2 infection. J Med Virol 2020;(epub ahead of print). https://doi.org/10.1002/jmv.25897
62. Stockman LJ, Bellamy R, Garner P. SARS: Systematic review of treatment effects. PLoS Med 2006;3(9):1525-1531. https://doi.org/10.1371/journal.pmed.0030343
63. Rodrigo C, Leonardi-Bee J, Nguyen-Van-Tam J, et al. Corticosteroids as adjunctive therapy in the treatment of influenza. Cochrane Database Syst Rev 2016;2016(3). https://doi.org/10.1002/14651858.CD010406.pub2
64. Delaney JW, Pinto R, Long J, et al. The influence of corticosteroid treatment on the outcome of influenza A(H1N1pdm09)-related critical illness. Crit Care 2016;20(1):75. https://doi.org/10.1186/s13054-016-1230-8
65. Aoki FY, Macleod MD, Paggiaro P, et al. Early administration of oral oseltamivir increases the benefits of influenza treatment. J Antimicrob Chemother 2003;51(1):123-129. https://doi.org/10.1093/jac/dkg007
66. Murphy BR, Baron S, Chalhub EG, et al. Temperature-sensitive mutants of influenza virus. IV. Induction of interferon in the nasopharynx by wild-type and a temperature-sensitive recombinant virus. J Infect Dis 1973;128(4):488-493. https://doi.org/10.1093/infdis/128.4.488

*Accepted 24 April 2020.*
